# Pituitary Apoplexy Complicated by Pulmonary Embolism in a Neurologically Complex Patient: A Diagnostic Challenge

**DOI:** 10.7759/cureus.118537

**Published:** 2026-10-24

**Authors:** Gergis Farag, Abdullatif Farhat

**Affiliations:** 1 Respiratory Medicine, The Grange University Hospital, Aneurin Bevan University Health Board, Cwmbran, GBR; 2 Geriatric Medicine, Royal Gwent Hospital, Aneurin Bevan University Health Board, Newport, GBR

**Keywords:** headache, hypopituitarism, magnetic resonance imaging, peripheral visual field defect, pituitary adenoma, pituitary apoplexy

## Abstract

Pituitary apoplexy is a rare endocrine emergency resulting from either acute haemorrhage or infarction within the pituitary gland. Although apoplexy occurs most frequently in a pre-existing pituitary adenoma, apoplexy could be the first presentation of a previously unknown adenoma. Acute headaches, visual disturbances, and neurological symptoms are common presenting symptoms in apoplexy; however, these symptoms may overlap with other neurological emergencies, which necessitate a holistic approach of clinical, radiological, and hormonal evaluation for suspected cases of apoplexy to confirm the diagnosis. Here we present a diagnostically challenging case of a 66-year-old man who presented with a sudden severe headache. His background history included a presumed intraventricular neurocytoma under surveillance. Initial CT head scan suggested possible haemorrhage within a known intraventricular mass (neurocytoma) with no obvious changes within the pituitary gland. However, subsequent MRI head demonstrated radiological features consistent with pituitary apoplexy. Later in his admission, he exhibited bilateral peripheral visual field constriction and early papilledema on fundoscopy. Hormonal testing showed evidence of hypopituitarism. The patient was managed conservatively with intravenous hydrocortisone replacement. During admission, he developed bilateral pulmonary emboli, for which he was treated with anticoagulation. While he was on anticoagulation, he developed intracranial haemorrhage, which led to his death after discharge from the hospital. This case demonstrates the importance of considering pituitary apoplexy in a challenging radiological and clinical presentation. A pre-existing neurological lesion (neurocytoma) makes a holistic approach, including MRI imaging and early hormonal evaluation are essential for timely diagnosis and treatment of pituitary apoplexy. The clinical challenge of having concurrent haemorrhagic and thrombotic events necessitates an individualised approach after balancing the benefits and risks of using anticoagulation treatment.

## Introduction

Pituitary apoplexy is a rare endocrine emergency caused by either haemorrhage within the pituitary gland or death of an area of the gland (infarction). Although most cases occur in pre-existing benign pituitary adenomas, most patients experience pituitary apoplexy as a first presentation of their underlying adenoma [1]. Most studies tell us that the incidence of apoplexy in pituitary adenoma is between 2-7% and 80% of the cases; apoplexy was the initial presentation of the condition [2,3]. Pituitary apoplexy usually presents with a variety of different symptoms such as acute headache, visual impairment (visual field defect, ocular palsies, diplopia and reduced visual acuity), neurological deficits, and adrenal insufficiency [4]. The diagnosis can be challenging as other neurological conditions can present with symptoms similar to apoplexy, such as intracranial haemorrhage, bacterial meningitis and cavernous sinus thrombosis. Apoplexy diagnosis should be considered in the emergency setting for any patient with acute neurological presentation, such as acute headache, especially when associated with visual disturbances, as any delay in the diagnosis can lead to permanent visual impairment or adrenal crisis.

A holistic approach including a detailed history focusing on the adrenal insufficiency symptoms and visual disturbances, endocrinological evaluation and an urgent MRI should be considered in any patient with suspected pituitary apoplexy [5]. The pathophysiology of apoplexy is not fully understood but there are many theories have been proposed to explain the mechanism of apoplexy including: (1) bleeding within growing pituitary adenoma due to vascular abnormality or inflammatory process; (2) sudden rise in pituitary perfusion driven by elevated blood pressure or increased intracranial pressure; (3) mismatch between blood supply and blood flow demand in a growing adenoma leading to ischemic necrosis within the gland; (4) reduced pituitary blood flow secondary to systemic hypoperfusion such as systemic shock; (5) increased hormonal stimulation of the pituitary gland similar to what happens in Sheehan’s syndrome [6-9]. Hypopituitarism is a common complication of pituitary apoplexy: a sudden rise in intrasellar pressure can compromise blood flow through the portal vessels and lead to subsequent anterior pituitary necrosis and reduction in hormonal release [9]. Adrenocorticotropic hormone (ACTH) deficiency is particularly important in the acute setting as reduced cortisol secretion can lead to haemodynamic instability and adrenal crisis. Thyroid and gonadal hormone deficiencies may also occur. MRI is the gold standard modality in the diagnosis of pituitary apoplexy due to its high sensitivity of around 88% in detecting the pituitary haemorrhage [10]. MRI can provide information about the haemorrhage, the underlying tumour and its relation to the surrounding anatomy [11]. Although CT is the most commonly used modality in acute settings, its low sensitivity of 21-46% makes it inferior to MRI in detecting apoplexy [12]. The choice between conservative management versus surgical intervention in pituitary apoplexy is still an ongoing topic of debate, and although there's a lack of consensus on this matter, medical treatment with steroids should be started for any patients with suspected pituitary apoplexy and showing any signs of adrenal insufficiency, pending radiological and laboratory confirmation. The subsequent decisions on the management plan should be discussed in a multidisciplinary team (MDT) on an individual basis. Pulmonary embolism (PE) is a serious life-threatening condition and is considered one of the most common causes of cardiovascular death.

The pathophysiology of PE is explained through Virchow’s triad and is a combination of venous stasis, endothelial injury, and a hypercoagulable state [13]. Many risk factors have been identified to contribute to PE, including acquired and inherited factors such as immobilisation, recent major surgery or trauma, active cancer, and antiphospholipid syndrome [14]. The risk of venous thromboembolic events in pituitary adenoma and apoplexy has not been explored thoroughly; a multicentre cohort study revealed increased risk of venous thromboembolic events in Cushing’s disease (a subtype of ACTH-secreting pituitary adenoma) [15].

## Case presentation

A 66-year-old male attended the acute medical unit complaining of a sudden-onset frontal headache that started one day before presentation; he presented with persistent, dull, aching, diffuse pain across the forehead. There were no associated visual disturbances on presentation, with no photophobia, vomiting, or focal neurological symptoms. The patient had a complex neurological history of an intraventricular mass (presumed neurocytoma) under surveillance, for which he previously underwent a partial surgical resection complicated by a post-operative haematoma and residual left-sided hemiparesis. His past medical history included a provoked PE, which was treated with anticoagulation for six months. His examinations were unremarkable for any new neurological or visual findings, although later during his admission he exhibited bilateral peripheral visual field constriction and early papilledema on ophthalmoscopic examination. Due to his significant neurological background and his presenting symptoms, a mass effect of his previously known intraventricular mass or intracranial bleeding were thought to be the cause of his symptoms and that's why a CT head was promptly done, which showed a trace of high density within the known intraventricular mass (presumed neurocytoma) raising concern for possible bleeding within the mass with no changes detected within the pituitary gland; however, a multidisciplinary meeting including neurosurgery and neuroradiology came with a conclusion that these findings aren't enough to confirm a bleeding within the mass and recommended an MRI for detailed assessment. On day 3 of symptoms onset, a subsequent MRI showed haemorrhagic changes in the pituitary gland and ruled out any haemorrhage within the intraventricular mass (Figure 1).

**Figure 1:**
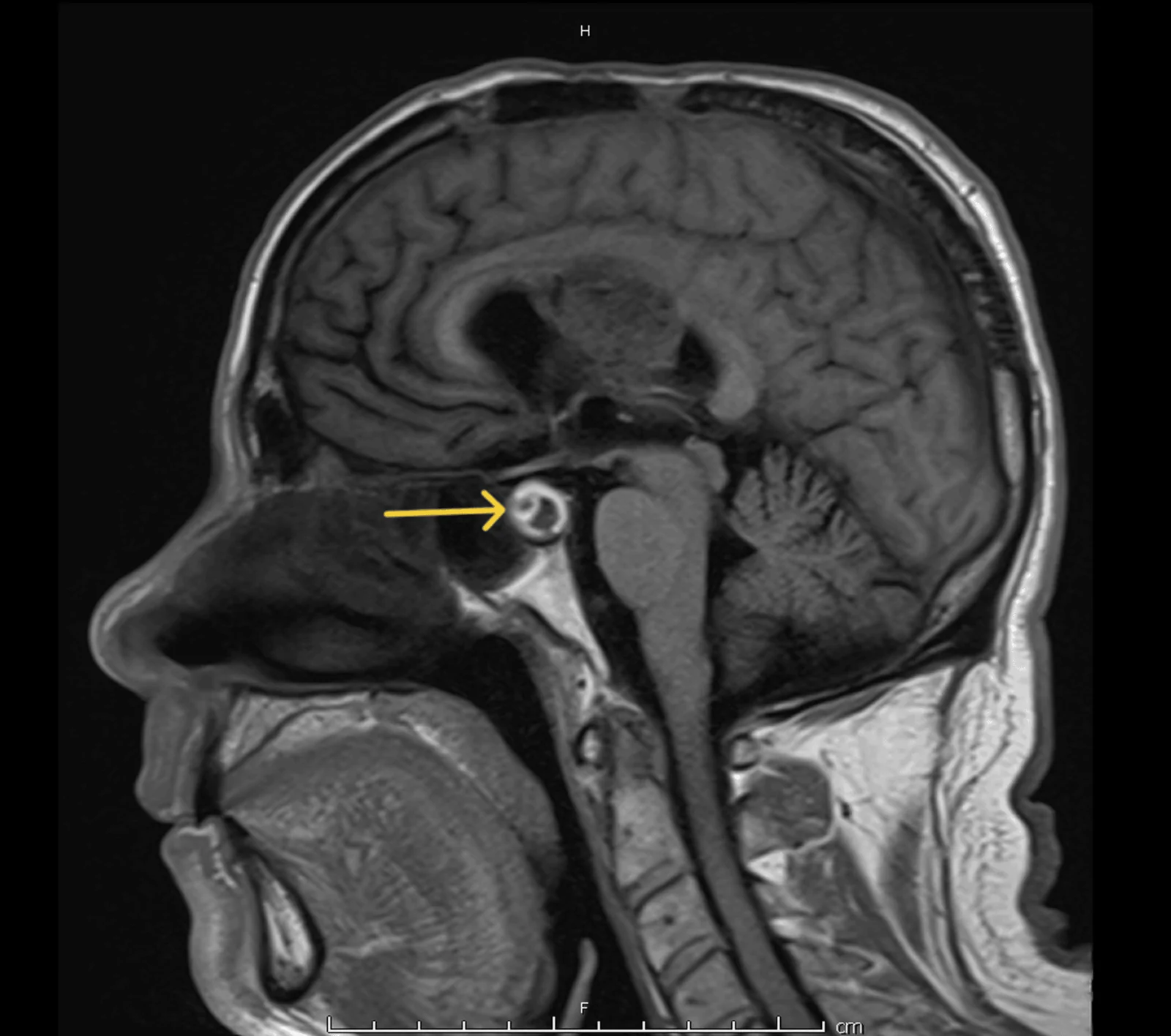
Sagittal T1-weighted MRI of the head demonstrating an enlarged, partially cystic pituitary gland with intrinsic T1 hyperintensity (yellow arrow), suggestive of pituitary apoplexy.

The diagnosis of pituitary apoplexy was based on clinical, laboratory, and radiological findings. Endocrine evaluation showed markedly reduced serum cortisol concentrations (53 nmol/L), low-normal gonadotrophin levels, and mildly suppressed thyroid-stimulating hormone, which raised concerns of pituitary dysfunction (Table 1). An ophthalmoscopic examination was done and showed peripheral visual field constriction. The standard MRI brain confirmed the presence of haemorrhagic changes within the pituitary gland, ruling out other neurological diagnoses.

**Table 1:** Baseline pituitary panel and serum electrolytes and post-hydrocortisone laboratory tests.

Test	Results	Normal Reference
Baseline laboratory tests		
Cortisol	53 nmol/L	Cortisol
Follicle-stimulating hormone	4.8 IU/L	1.4-18.1 IU/L
Luteinizing hormone	2.4 IU/L	1.5-9.3 IU/L
Thyroid-stimulating hormone	0.35 mU/L	0.55-4.78 mU/L
Thyroxine	16.1 pmol/L	11.5-22.7 pmol/L
Oestrogen	156.5 pmol/L	<146.0 pmol/L
Prolactin	70 mU/L	45-375 mU/L
Testosterone	11.6 nmol/L	6.5-23.7 nmol/L
Insulin-like growth factor-1	57.9 nmol/L	4.2-24.9 nmol/L
Potassium	2.9 mmol/L	3.5-5.0 mmol/L
Sodium	143 mmol/L	133-146 mmol/L
Post-hydrocortisone laboratory tests		
Cortisol	839 nmol/L	-
Potassium	4.1 mmol/L	3.5-5.0 mmol/L

Putting all together, the diagnosis of pituitary apoplexy was made and started immediately on IV hydrocortisone. An MDT of neurosurgery, neuroradiology, and endocrinology was involved in the management of the patient. The patient was managed conservatively with medical treatment of IV hydrocortisone 100 mg every six hours and regular monitoring of his hormonal and electrolyte disturbances. The patient showed improvement of his clinical symptoms and hormonal disturbances after receiving IV hydrocortisone. During hospital admission, our patient developed shortness of breath and an increase in his oxygen requirements, which was thought to be hospital-acquired pneumonia; after showing poor response to antibiotics, a CT pulmonary angiography (Figure 2) was done and revealed a PE.

**Figure 2:**
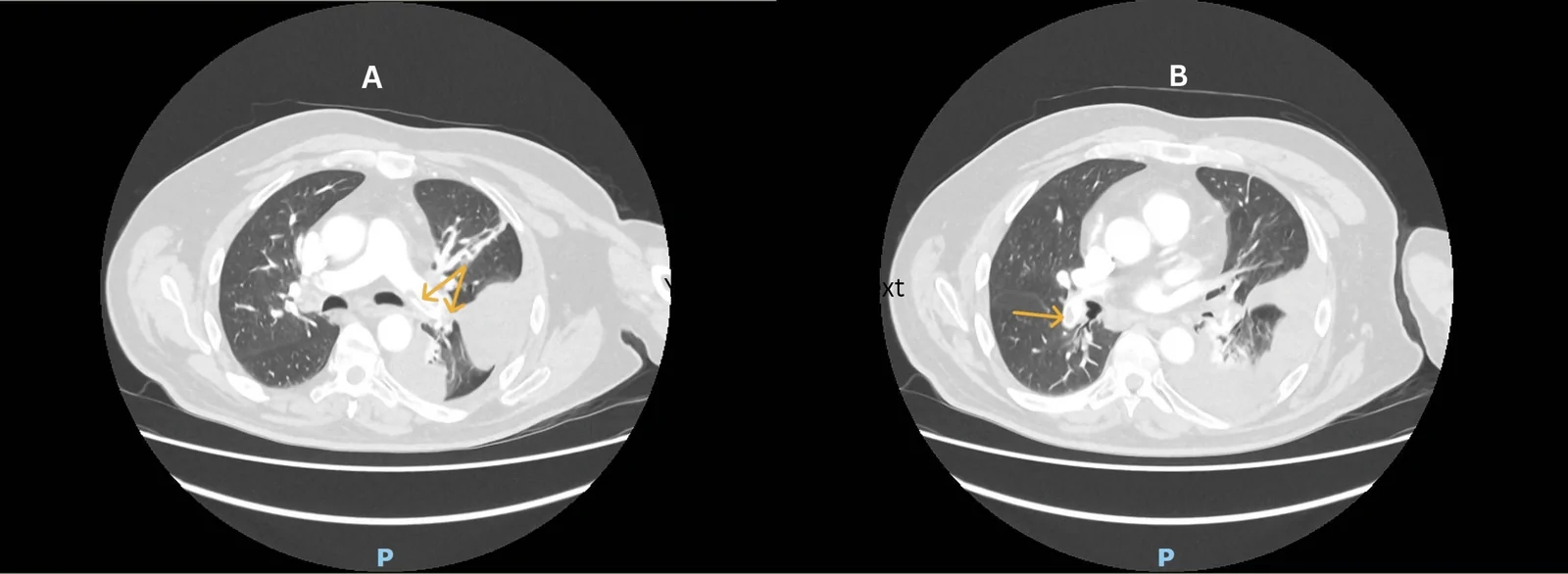
CT pulmonary angiography showing pulmonary emboli. (A) Axial cross-sectional image demonstrating emboli in the left pulmonary artery (yellow arrows). (B) Axial cross-sectional image demonstrating an embolus in the right pulmonary artery (yellow arrow).

Having concomitant thrombotic (PE) and haemorrhagic (pituitary apoplexy) events complicated the therapeutic management. Following a careful assessment of risks versus benefits of starting therapeutic anticoagulation after having a recent pituitary haemorrhage, the patient was commenced on low-molecular-weight heparin to treat a life-threatening PE. The decision was driven by many factors favouring commencement of anticoagulation versus factors opposing its use (Table 2). Therapeutic anticoagulation with low-molecular-weight heparin was selected over direct oral anticoagulants (DOACs) due to its effective short half-life.

**Table 2:** Factors considered favouring and opposing the use of anticoagulation therapy following pulmonary embolism in the setting of recent apoplexy.

Factors favouring anticoagulation	Factors opposing anticoagulation
Acute large-volume bilateral pulmonary embolism with risk of haemodynamic compromise	Recent pituitary haemorrhage with risk of recurrence or expanding intracranial bleeding
Interval of approximately three weeks since apoplexy diagnosis	Previous history of post-operative haematoma
No evidence of ongoing neuro-ophthalmic deterioration	Underlying intracranial lesion (neurocytoma)
Neuroimaging revealed a haemorrhage confined to the pituitary gland with no extensive involvement	Limited evidence to guide use of anticoagulation in recent pituitary apoplexy
Morbidity and mortality risk of untreated pulmonary embolism outweighed risk of apoplexy recurrence	-
Selection of low-molecular-weight heparin allowed greater control of anticoagulation effect reversibility if bleeding occurred.	-

Clinical signs of pituitary apoplexy deterioration, including neuro-ophthalmic symptoms such as severe headache, visual field defect, reduced visual acuity, altered level of consciousness and cranial nerve palsies, in addition to worsening hypopituitarism, were monitored closely. The patient improved clinically and was discharged with a plan to have a dedicated pituitary phase MRI after discharge. The patient had an unfortunate event of new intracranial haemorrhage while on anticoagulation 14 days after discharge, leading eventually to his death. A timeline for the sequence of events since the symptom onset is shown in Table 3.

**Table 3:** Chronological summery of clinical events.

Event	Day
Sudden severe headache	0
CT showed possible bleeding within known intraventricular mass	1
MRI demonstrated pituitary apoplexy	3
Started on IV hydrocortisone	4
CT pulmonary angiography demonstrated bilateral pulmonary embolism	21
Multi-disciplinary team decision regarding commencing anticoagulation	21
Discharge from the hospital after clinical improvement	33
Developing new neurological deterioration and CT demonstrated intracranial haemorrhage	14 days after discharge

## Discussion

Pituitary apoplexy remains a diagnostic challenge, although it is considered an endocrine emergency that can lead to serious complications. The overlap of its symptoms with other neurological conditions makes it often difficult to detect early [5]. Severe headache is the main presenting symptom; according to the International Classification of Headache Disorders (ICHD-3), headache attributed to pituitary apoplexy is described as severe, sudden onset and associated with visual symptoms and/or hypopituitarism [15,16]. Headache is a common symptom in several neurological conditions and emergencies such as intracranial haemorrhage, where headache is described as sudden or thunderclap onset, may be localised to the site of intracerebral haemorrhage and associated with focal neurological signs [16]. The diagnostic complexity in our patient arises from having a pre-existing intracranial lesion (presumed neurocytoma), which directed attention initially towards a mass effect caused by the lesion or bleeding within this lesion. The interpretation of headache in correlation to other symptoms is essential to avoid missing a serious diagnosis; the presence of visual symptoms and hypopituitarism was pointing towards pituitary apoplexy in this case.

CT has a low sensitivity in diagnosing apoplexy, and it may not detect pituitary apoplexy, especially in patients without a pre-existing adenoma [12]. MRI has a great diagnostic accuracy in pituitary apoplexy patients and can be used to rule out other differential diagnoses. It is considered the gold standard in the diagnosis [5]. In our case, MRI ruled out any bleeding within the known intraventricular lesion and showed an enlarged, partially cystic pituitary gland with some intrinsic T1 hyperintensity consistent with pituitary apoplexy features on MRI. The CT findings weren't helpful in directing the diagnosis to the pituitary pathology due to its limited sensitivity and the absence of a known history of pituitary adenoma in the patient. Pituitary apoplexy occurs most of the time in a pre-existing adenoma. However, in some cases, apoplexy can be the initial presentation of the symptoms. Qureshi and Thozhuthumparambil reported a case presenting with apoplexy as a first manifestation of pituitary macroadenoma in a male patient in his early 60s; due to the initial presentation of headache, meningism and fever, it was thought understandably to be a case of meningitis, but subsequent investigations ruled out any CNS infection or subarachnoid haemorrhage and the diagnosis of apoplexy was confirmed with an MRI head scan [17]. Presenting with apoplexy as an initial symptom of pituitary adenoma occurred in 80% of pituitary adenoma cases that developed apoplexy [2,3]. The neurological background of the patient, the absence of any abnormalities within the pituitary gland on the initial CT, and the absence of a pre-existing adenoma made the diagnosis and management of this case challenging and highlighted the importance of a holistic approach when treating patients with acute headache, especially when visual disturbances are present. The triad of clinical findings of headache and neuro-ophthalmic signs, hormonal evidence of pituitary insufficiency in the form of secondary adrenal insufficiency, and the MRI findings of pituitary haemorrhage should be used in the management of pituitary apoplexy cases [5].

Various factors have been identified as precipitating factors in pituitary apoplexy, including medications (anticoagulation and antiplatelets), major surgeries (coronary artery bypass graft), head trauma, growth hormone (GH)- and ACTH-secreting pituitary adenomas, diabetes, hypertension, and severe hypotension and shock [3]. The patient described in this report has several risk factors that might have contributed to his condition, including hypertension, recent neurosurgical operation, and interventricular neurocytoma. The association between pituitary apoplexy and neurocytoma is rare; that’s why we couldn’t find a clear pathogenesis of this association in the literature. Soh et al. reported a case of A 48-year-old male who presented with an 11-month history of syncopal episodes and questionable seizures; subsequent CT and MRI revealed a mass that proved to be a central neurocytoma and a suprasellar mass consistent with pituitary macroadenoma [18]. Although the case didn’t present with apoplexy, it highlighted the association between pituitary adenoma and neurocytoma.

Acute adrenal insufficiency is identified as one of the serious complications of pituitary apoplexy, and it occurs secondary to disruption of the release of ACTH from the pituitary gland [15]. It can lead to serious consequences and cardiovascular failure if not treated early, so empirical treatment with glucocorticoids (IV hydrocortisone) should be considered for all patients with suspected pituitary apoplexy who are showing signs of adrenal insufficiency [5].

A challenge in the management of this patient arose when our patient developed a PE while admitted to the hospital. The occurrence of a thrombotic event shortly after pituitary haemorrhage made the treatment decision more complicated. An MDT decision was taken to start the patient on anticoagulation after balancing risks versus benefits, aiming to treat a life-threatening PE and avoid aggravating a pituitary haemorrhage. Lee and Fang reported a similar sequence in a patient who developed massive PE 18 days after intracerebral haemorrhage. Their patient received unfractionated heparin followed by rivaroxaban without recurrent intracranial bleeding during follow-up [19]. Although the site of haemorrhage differed, the case illustrates the similar challenge in treating PE when further bleeding is a concern. Our patient received low-molecular-weight heparin due to its shorter half-life compared to DOACs and greater reversibility, allowing rapid reversal of the anticoagulation effect if recurrent pituitary haemorrhage occurred. There are no guidelines for treating concomitant PE and pituitary apoplexy, and the treatment should be selected according to the individual circumstances of each case after balancing risks versus benefits.

## Conclusions

This case highlighted the importance of considering pituitary apoplexy in any patient presenting to the emergency department with acute headache, especially if this was accompanied by visual symptoms and hormonal abnormalities. It has been demonstrated in this case that having a pre-existing intracranial lesion (neurocytoma) shouldn't distract from maintaining a broad differential diagnosis, and neurological symptoms should be analysed in the context of laboratory and radiological findings. The symptomatic overlap between different neurological emergencies should prompt clinicians to investigate the condition early using an appropriate modality, with MRI considered the cornerstone in the diagnosis of pituitary apoplexy. Most of the cases of pituitary apoplexy present in a pre-existing pituitary adenoma, while those presenting in a non-pre-existing adenoma remain a diagnostic challenge, especially in a patient with a background of a neurological lesion (neurocytoma). Empirical treatment with glucocorticoids is the cornerstone in medical management of pituitary apoplexy complicated by adrenal insufficiency. The case illustrates the therapeutic dilemma of treating concurrent thrombotic and haemorrhagic events and emphasises the importance of an MDT approach and patient-centred decision-making that balances risks versus benefits of therapeutic decisions. Despite multidisciplinary management and careful assessment, the patient suffered intracerebral haemorrhage while on anticoagulation, highlighting the complexity of balancing risks and benefits of using anticoagulation in a complex patient.
